# Phosphoinositides and inositol phosphates as molecular glues

**DOI:** 10.1002/1873-3468.70388

**Published:** 2026-06-19

**Authors:** Aleshia Seaton‐Terry, Alexander J. Cutright, Mong Na Claire Loi, Jessica Martin, Joseph W. Gilligan, Jakub N. Kubina, Michael J. Arambula, Susanna Tanada Palmu, Daniella Zhou, Raymond D. Blind

**Affiliations:** ^1^ Division of Diabetes, Endocrinology and Metabolism, Department of Medicine Vanderbilt University Medical Center Nashville TN USA; ^2^ Department of Biochemistry and Pharmacology Vanderbilt University School of Medicine Nashville TN USA

**Keywords:** allosteric regulation, cell signaling, enzymatic regulation, inositol phosphate signaling, lipid–protein interactions, molecular glue mechanisms, phosphoinositide signaling, signal transduction, structural biology

## Abstract

Inositol phosphates (IPs) and phosphoinositide lipids (PIPs) are regulatory molecules critical for a wide array of functions in eukaryotic cells. Membrane PIPs have clear functions in transient recruitment of signaling proteins to membranes, and IPs have been found locked in the core of proteins as structural cofactors. However, several recent studies have suggested IPs and PIPs can mediate protein–protein interactions at the interface between proteins, functioning as natural molecular glues. Here, we present recent structural biology describing how IPs and PIPs mediate these regulatory responses at protein interfaces. In addition, we describe protein–protein interactions mediated by IPs and PIPs, for which evidence supporting a natural molecular glue role is unclear or awaits further high‐resolution structural analyses. Together, we put forth that PIPs and IPs have a historically under‐appreciated role at protein–protein interfaces, requiring a more systematic, structural approach to elucidate.

## Graphical abstract

Inositol phosphates (IPs) and phosphoinositides (PIPs) regulate diverse eukaryotic processes. Beyond recruiting signaling proteins or acting as structural cofactors, recent studies suggest they mediate protein–protein interactions as natural molecular glues. We review structural evidence for IP/PIP‐mediated interfaces, highlight unresolved cases requiring high‐resolution analyses, and propose that these molecules play an under‐appreciated regulatory role at protein interfaces.

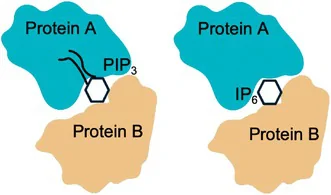

## Abbreviations

**ARP2/3**, Actin‐related protein 2/3 complex

**cGAMP**, Cyclic guanosine monophosphate‐adenosine monophosphate

**CTD**, C‐terminal domain

**DAD**, Deacetylase‐activation domain

**HDAC**, Histone deacetylase

**IP**_**6**_, Inositol hexakisphosphate

**IP**_**7**_, 5‐diphosphoinositol pentakisphosphate

**IPs/InsPs**, Inositol phosphates

**NTSR1**, Neurotensin receptor 1

**PIP**_**2**_, Phosphatidylinositol 4,5‐bisphosphate

**PIP**_**3**_, Phosphatidylinositol 3,4,5‐trisphosphate

**PIPs**, Phosphoinositides

**SF‐1m**, Steroidogenic factor‐1

**βarr1**, β‐arrestin 1

Inositol phosphates (IPs) and phosphoinositides (PIPs) comprise two major families of inositol‐based signaling molecules that differ in charge, localization, and enzymatic regulation. Compared with PIPs, IPs have more positions that can be phosphorylated, often giving them a higher negative charge density than even the most highly phosphorylated PIP, PIP_3_. IPs are water‐soluble molecules and can use their dense, negative charge for electrostatic interactions with proteins [1], sometimes functioning as an ‘inositol phosphate code’ [2, 3, 4, 5, 6, 7]. In contrast, PIPs are membrane‐anchored lipids generated by phosphorylation of phosphatidylinositol, creating a variety of distinct species (e.g., PIP, PIP_2_, PIP_3_, among others) that are usually spatially restricted to specific membranes [8, 9]. Their metabolism is rapid and tightly controlled by lipid kinases, phosphatases, lipases and other enzymes that permit transient signaling events associated with membranes [10]. Together, these two classes of small molecules generally operate in different cellular compartments (membranes vs. cytosol/nucleus) but share a common *myo*‐inositol chemical core. The soluble nature of IPs allows them to freely diffuse and interact with proteins [11, 12]; by the same token, PIPs are membrane‐anchored, with any movement away from membranes restricted by the acyl chains [13]. As we shall see, there are several examples where PIPs indeed leave the membrane bilayer and can be detected in the soluble nucleoplasm or cytoplasm [14].

**Fig. 1:**
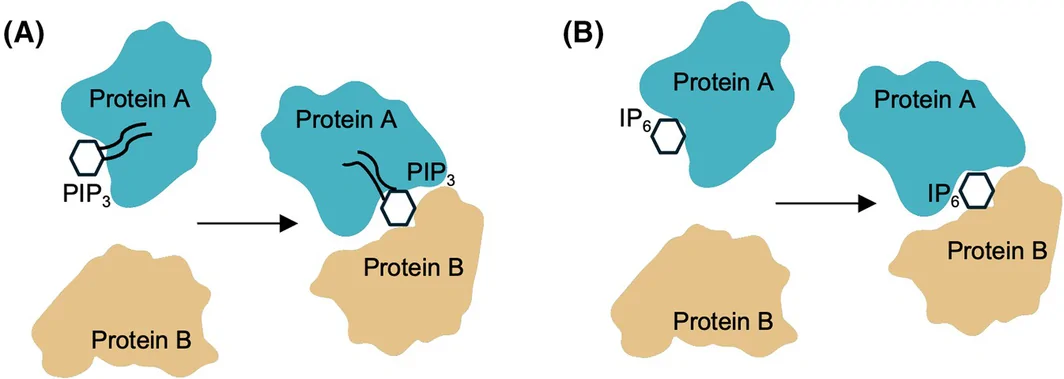
Representative illustration of a formation of a quaternary complex by a molecular glue. (A) The PIP_3_ acts as the interface between the two proteins with the head group and tail group binding to distinctive binding regions in each protein, acting as the ‘glue’ of the complex. (B) Representative illustration of a formation of two abstract proteins A, and B, into a quaternary complex by IP_6_. These schematics show that PIP_3_ and IP_6_ act as molecular glues by interfacing two proteins.

**Fig. 2:**
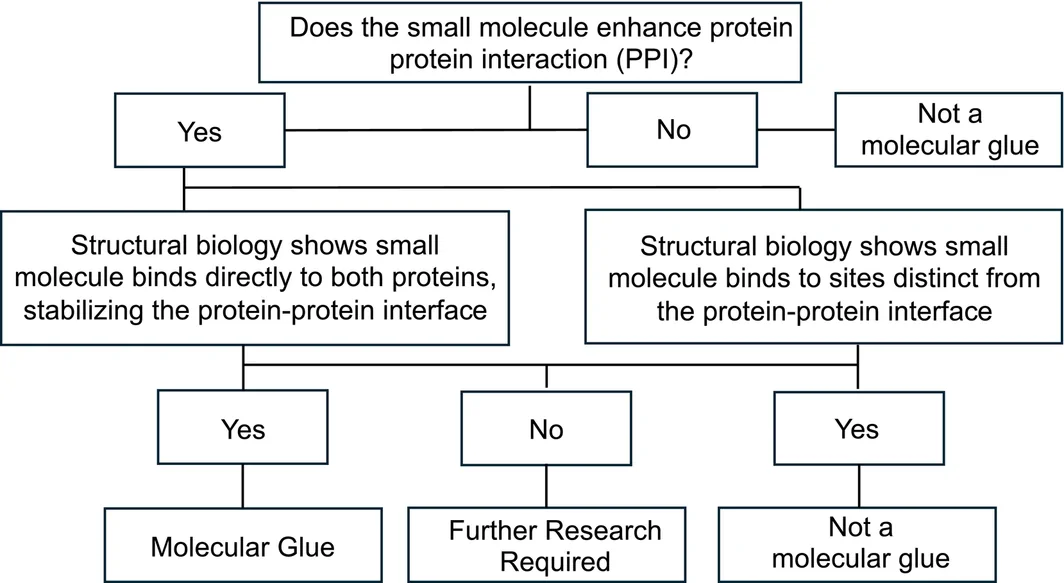
Decision tree for classifying molecular glues. This figure illustrates a stepwise framework for determining whether a small molecule functions as a molecular glue. The process begins by asking whether the molecule enhances or induces protein–protein interactions. Subsequent steps evaluate whether the molecule binds directly to both proteins at the interface or binds to sites distinct from the protein–protein interface. Outcomes include confirmed molecular glue, nonmolecular glue (representing classical allosteric or cofactor effects), or further research required.

IPs and PIPs play diverse roles in eukaryotic cells, extending from fundamental cellular processes, such as gene expression and metabolism to crucial physiological functions, such as development and immune responses [15, 16, 17, 18, 19]. As part of the diverse range of mechanisms, PIPs and IPs are increasingly recognized for their remarkable ability to act as ‘molecular glues’ [14]. Beginning this analysis, it is important to differentiate between the concepts of a molecular glue *vs*. a conventional allosteric small molecule mechanism of action. A molecular glue is a small molecule that stabilizes the interaction between two proteins (or protein domains) by binding at the protein–protein interaction interface, while allosteric mechanisms rely on small molecule‐induced protein conformational changes, where the small molecules bind at a site distinct from the protein–protein interface (Figs 1 and 2). In this way, high‐resolution structural information about the precise location of small molecule interaction sites (either in the protein–protein interface or distal to the interface) is critical to defining a small molecule as a molecular glue. Here, we are specifically excluding synthetic systems, such as proteolysis‐targeting chimeras (PROTACs), or pharmacological molecular glues, such as thalidomide [20], which tether an E3 ligase to a target protein at the interface. We exclude those molecules from this analysis because they are synthetic, whereas this review focuses on natural molecular glues, further restricted to the IPs and PIPs. Regardless, any molecular glue mechanism, natural or synthetic, is distinct from allosterically modulated mechanisms of protein–protein interaction [21].

**Fig. 3:**
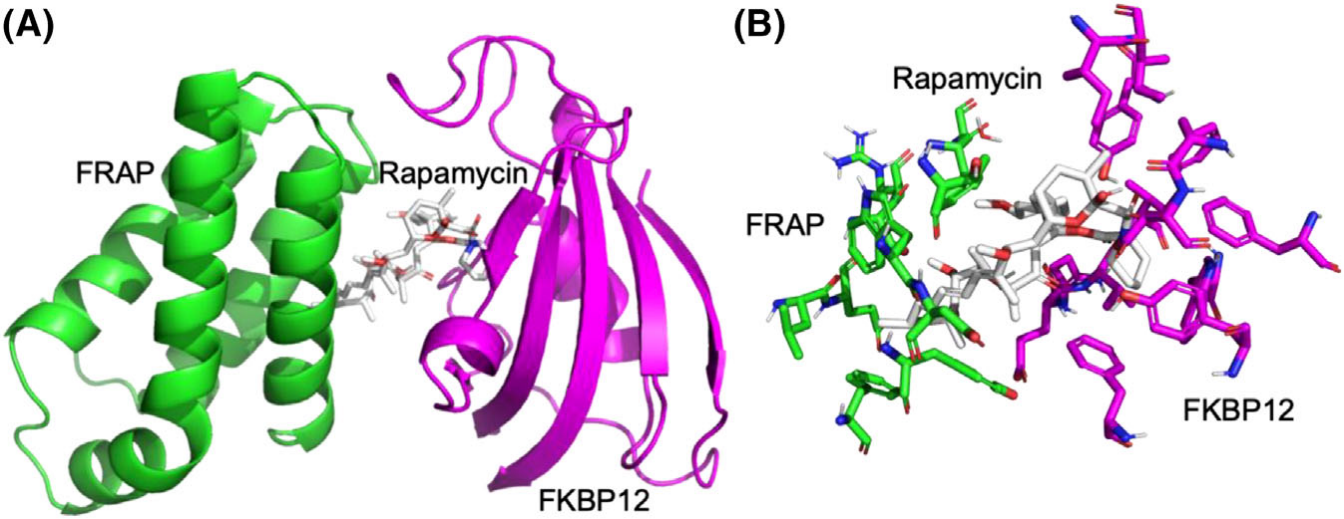
Rapamycin is an established molecular glue that mediates the interaction between FKBP12 and FRAP. (A) The well‐known molecular glue interaction between FRAP (green) and FKBP12 (magenta) mediated by the small molecule rapamycin (stick) (PDB: 1FAP), and (B) the magnification of the interface between FRAP (green) and FKBP12 (magenta) by rapamycin (sticks) (PDB: 1FAP). These data suggest that within well‐characterized systems, molecular glues exist in the interface between the target proteins, serving to drive protein–protein interactions at those interfaces.

There are several classically studied examples of molecular glues that, although do not involve an IPs or PIPs, deserve mentioning. One of the most well‐characterized examples is the FKBP12‐rapamycin‐mTOR system (Fig. 3). In this system, rapamycin, an immunosuppressive natural product from bacteria, simultaneously binds to the hydrophobic binding pocket of FK506 binding protein (FKBP12) and FKBP‐rapamycin‐associated protein (FRAP), also known as the target of rapamycin (mTOR) to stabilize their interaction and modulate downstream signaling [22]. Serine 2035 within the FKBP12‐rapamycin binding domain (FRB) in FRAP is crucial for the interaction between FRAP and FKBP12‐rapamycin [23]. Tryptophan 2027 is also responsible for the FKBP12–rapamycin–mTOR complex [24]. Rapamycin interacts with FKBP12 by forming three hydrogen bond interactions with E54, I56 and Y82 and hydrophobic contacts with Y26, F36, F46, V55, I56, W59, Y82, I90, I91 and F99 [25]. The structure of the FKBP12–rapamycin–mTOR complex (Fig. 3) consists of a β sheet made of five antiparallel β strands with a short α helix. Rapamycin binds in a hydrophobic pocket formed between the α helix and the β sheet FKBP12 while also engaging a hydrophobic pocket within the FRB domain of mTOR, which is comprised of helices α1 and α4 [26]. This tertiary complex is stabilized by hydrophobic interactions between rapamycin and specific residues of the mTOR FRB domain, specifically with W2101, Y2104, Y2105, F2108, L2031, and F2039 [25]. The extensive characterization of rapamycin proves the principle of molecular glue as a regulatory mechanism in biology.

A second example that is well‐characterized and does not involve IPs or PIPs is the plant hormone auxin, which mediates the interaction between the E3 ubiquitin ligase TIR1 and AUX/IAA transcriptional repressors. The function of this interaction is to promote AUX/IAA degradation, which regulates plant growth [27]. Structural studies have clearly shown auxin in the interface between these two proteins [27], and without auxin, the interaction between these proteins is less stable [27]. In addition to these examples, adenosine monophosphate has been shown to act as a molecular glue between ChREBP and the 14‐3‐3 adaptor protein [28], which has inspired the development of novel synthetic molecules to protect pancreatic beta cells [28]. Together, these examples of molecular glues prove the principle that endogenous small molecules can act as mediators of protein complex assembly across a wide variety of biological contexts. Furthermore, the distinction between a biological molecular glue and an allosteric mediator can often only be determined by structural biology, such that the precise position of the small molecule in the interface between two proteins or protein domains can be used to differentiate between allosteric mechanisms.

IPs and PIPs both achieve molecular glue activity by leveraging their phosphate groups to form intricate networks of interactions with basic and polar residues on protein surfaces, bridging two proteins or domains in a functional interaction [29]. PIPs can also use their acyl chains as one end of the glue functionality, which when the acyl chains are used as part of the molecular glue, requires a hydrophobic pocket or region in one of the two target proteins [14, 30]. This unique mode of action allows molecular glues to dynamically regulate protein–protein interactions without allosteric structural changes. In this way, molecular glues can alter protein interactions without disturbing protein structure or native functions while altering interaction partners. Here, we focus specifically on the molecular glue functions of IPs and PIPs.

## Significance of molecular glues

IPs and PIPs can function as interface‐bridging molecules, physically linking two or more proteins at their interaction surfaces [29]. This mode of regulation differs fundamentally from classical allostery, in which a small molecule induces conformational changes within a protein to regulate its interaction with other proteins or its enzyme activity. While IPs can stabilize complexes that would otherwise be short‐lived or fail to form, controlling signaling through this mechanism [12, 31] membrane‐localized PIPs further add compartment‐specific logic by restricting where complexes can form, which more directly couples signaling outputs to a particular cellular location [8, 32]. This dual ability to stabilize complexes and localize signaling provides a versatile framework for understanding how small molecules like IPs and PIPs function as interface‐bridging endogenous molecules, thus altering signaling (Box 1, Tables 1 and 2).

**Table 1:** Classification of inositol phosphates and phosphoinositides by compartment, evidence, and molecular glue activity.

Classification	Glue/pseudo‐glue	Protein 1	Protein 2	Primary compartment(s)	Evidence type(s)	PDB ID	References
Signaling	IP_6_	MTA1	HDAC1	Cytosol and nucleus	Structural, biophysical, genetics	5ICN	[33]
Signaling	IP_4_	SMRT‐DAD	HDAC3	Cytosol and nucleus	Structural, biophysical, genetics	4A69	[12]
Structural/assembly	IP_6_	HIV‐1 Gag (CA‐SP1 region)	HIV‐1 Gag (CA‐SP1 region)	Cytosol and nucleus	Structural, biophysical, genetics	6BHT	[31]
Signaling	PIP_3_	SF‐1	Coactivator proteins (ex.PGC1‐α)	Plasma membrane (inner leaflet)	Structural, biophysical, genetics	4QJR	[14]
Signaling	PIP_2_	NTSR1	β‐arrestin 1	Plasma membrane (inner leaflet)	Structural, biophysical, genetics	6UP7	[34]
Signaling	PIP_2_	Kir2.2 TMD	Kir2.2 CTD	Plasma membrane (inner leaflet)	Structural, biophysical, genetics	3SPI	[35]
Signaling	IP_6_	Arr2	Arr2 and Arr3	Cytosol and nucleus	Structural, biophysical, genetics	1ZSH	[36]
Signaling	PIP_2_ (PI(3,5)P2)	STING	STING	Endoplasmic reticulum membrane	Structural and genetics	9DAT	[37]
Signaling	PIP_2_ (PI(4,5)P2)	STING	STING	Endoplasmic reticulum membrane	Structural and genetics	9DAN	[37]
Signaling	IP_7_	PHO81	PHO85	Cytosol and nucleus	Biophysical and genetics	Proposed scheme	[38, 39]
Signaling	IP_8_	PHO81	PHO85	Cytosol and nucleus	Biophysical and genetics	Proposed scheme	[38, 39]

**Table 2:** Lifetime and modifiability of key inositol phosphates and phosphoinositides.

Molecule	Lifetime	Modifiability	References
IP_6_	Rapid turnover	Interconverted by IP_6_ kinases (IP_6_Ks)	[11, 40]
IP_4_	Rapid turnover	Phosphorylated and dephosphorylated by specific kinases and phosphatases	[11, 40]
IP_7_	Rapid turnover	Synthesized by IP_6_Ks; hydrolyzed by phosphatases	[11, 40]
PIP_2_	Rapid turnover	Hydrolyzed by PLC; phosphorylated to PIP_3_; dephosphorylated by lipid phosphatases	[8, 11]
PIP_3_	Short‐lived	Generated by PI3K; dephosphorylated by PTEN/SHIP	[8]

Box 1 **Inositol phosphates, phosphoinositides, and binding modes**

- Inositol phosphates (IPs/InsPs) are soluble molecules derived from myo‐inositol, numbered according to the positions of phosphate substitution on the inositol ring (e.g., IP₆ = InsP₆; IP₇ = diphosphoinositol pentakisphosphate).
- Phosphoinositides (PIPs) are membrane‐embedded lipids denoted by phosphorylation on the inositol headgroup (e.g., PIP_2_; PIP_3_).
- PH (pleckstrin homology) domains preferentially bind specific PIPs (commonly PIP_2_ or PIP₃) to mediate membrane recruitment rather than enzymatic activation.
- *Note:* See Table 1 (Glues) for context‐specific examples of interface stabilization.

## What doesn't count: Nonmolecular glue interactions

Although we will provide a few examples of molecular glues, the vast majority of characterized small molecule interactions with proteins do not regulate the bound proteins as molecular glues. Most prominent are small molecules that allosterically regulate protein functions. In this standard mode of regulation, the small molecule binds at a site distinct from a protein–protein interface, inducing a conformational change in the protein that induces fit between the two proteins. For example, cAMP activates protein kinase A (PKA) by binding to its regulatory subunits, inducing a conformational change that releases the catalytic subunits [41]. Although cAMP ultimately influences protein–protein interactions, it does so from a remote location, translating conformational changes throughout the protein rather than by bridging two proteins together at their interface [41]. Similarly, ubiquitous cofactors such as NAD^+^ or ATP often bind individual proteins to support catalysis but do not stabilize interactions between two distinct proteins by bridging the proteins together at a protein–protein interface [42].

These cases represent interaction modes that strongly regulate protein function or localization yet fail to meet the operational definition of a molecular glue, which requires the small molecule to exist in the interface and to stabilize that protein–protein interface. Accordingly, allosteric interactions induced by small molecules are classified as allosteric functions rather than interface‐bridging functions of the small molecule. In targeted protein degradation (TPD) drug design, molecular glues usually refer to synthetic small molecules that increase the action of an E3 ligase on a particular target protein, the classic example being thalidomide recruitment of neo substrates to cereblon [20]. Proteolysis‐targeting chimeras (PROTACs) are bifunctional molecules that mediate protein–protein interactions by physically linking two proteins via two engineered warheads designed to tether an E3 ligase to a target protein. Since these are synthetic engineered small molecules, they fall outside the scope of the naturally occurring endogenous molecular glues considered here [43].

## IP_4_ and IP_6_ in HDAC‐corepressor interactions

**Fig. 4:**
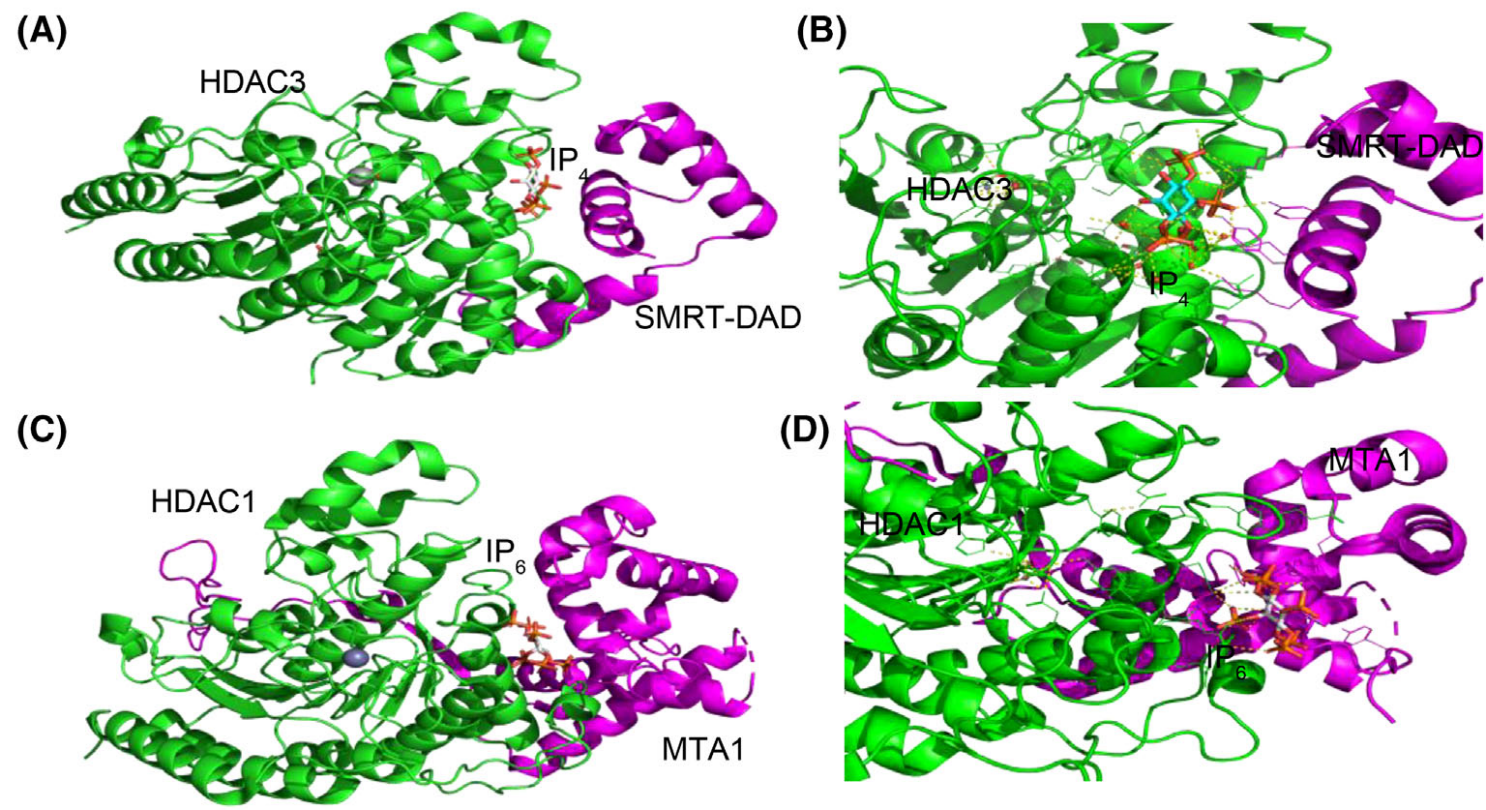
Inositol phosphates (IPs) exist in the interface between Class I histone deacetylases (HDACs) and corepressors and form interactions with both proteins at the interface. (A) Interaction of the SMRT‐DAD (magenta) with the HDAC3 (green) by IP_4_ (stick), and Zn (gray sphere) (PDB: 4A69), with (B) highlighting the ligand‐binding pocket with interactions shown with yellow dashed lines (PDB: 4A69). (C) The interaction of the MTA1 (magenta) with the HDAC1 (green) by IP_6_ (stick), and Zn (gray sphere) (PDB: 5ICN), and (D) highlighting the ligand‐binding pocket with interactions shown with yellow dashed lines (PDB: 5ICN). This shows the mechanism of interaction that is modulated by IP_6_.

The first instance of an IPs acting as a molecular glue is the D‐*myo*‐inositol‐(1,4,5,6)‐tetrakisphosphate, or Ins(1,4,5,6)P_4_, or simply IP_4_, which bridges HDAC3 and SMRT‐DAD [12]. The crystal structure of this complex shows IP_4_ binding within a highly basic pocket at the interface, physically linking the two proteins (Fig. 4A,B) [12]. In addition to enhancing HDAC enzyme activity through a glue‐independent mechanism, this IP has a glue‐like function in stabilizing the complex of HDAC3 and SMRT‐DAD, as the complex loses stability when the IPs' binding site is mutated, specifically amino acid R265 [12, 44]. *In vitro* reconstitution assays further confirm that IP_4_ uniquely promotes the assembly and activation of HDAC3 with SMRT‐DAD [12]. Together, these results suggest that IP_4_ can act as an endogenous molecular glue, essential for the structural integrity and catalytic activation of the HDAC3 corepressor complex, further illustrating how IPs can directly promote assembly and function at a protein–protein interface.

Similarly, structural studies of the HDAC1‐MTA1 complex revealed that IP_6_ can act as a molecular glue to stabilize the interaction, also resulting in enhanced enzymatic activity (Fig. 4C,D) [33]. This interaction is dependent on the number and precise positioning of phosphates on the inositol ring [33], and further work showed mutation of R270 results in a two‐fold reduction of HDAC1 activation [33, 44]. Together, these data suggest that HDAC1 and HDAC3 both use IPs as molecular glues to stabilize interactions with their respective transcriptional corepressors.

## IP_6_ in HIV‐Gag hexamer

The inositol phosphate IP_6_ is essential for the HIV‐1 life cycle, acting as a structural bridge that helps the virus build its protective shell during both assembly and maturation [31, 45, 46]. During assembly, IP_6_ facilitates the formation of the six‐helix bundle by binding to the two rings of primary amines at lysine residues K290 and K359 (which correspond to K158 and K227 in the mature capsid) [31, 47]. By coordinating these residues, IP_6_ neutralizes the repulsive electrostatic interactions at the center of the hexamer, acting as the glue that holds the immature lattice together [31, 47].

**Fig. 5:**
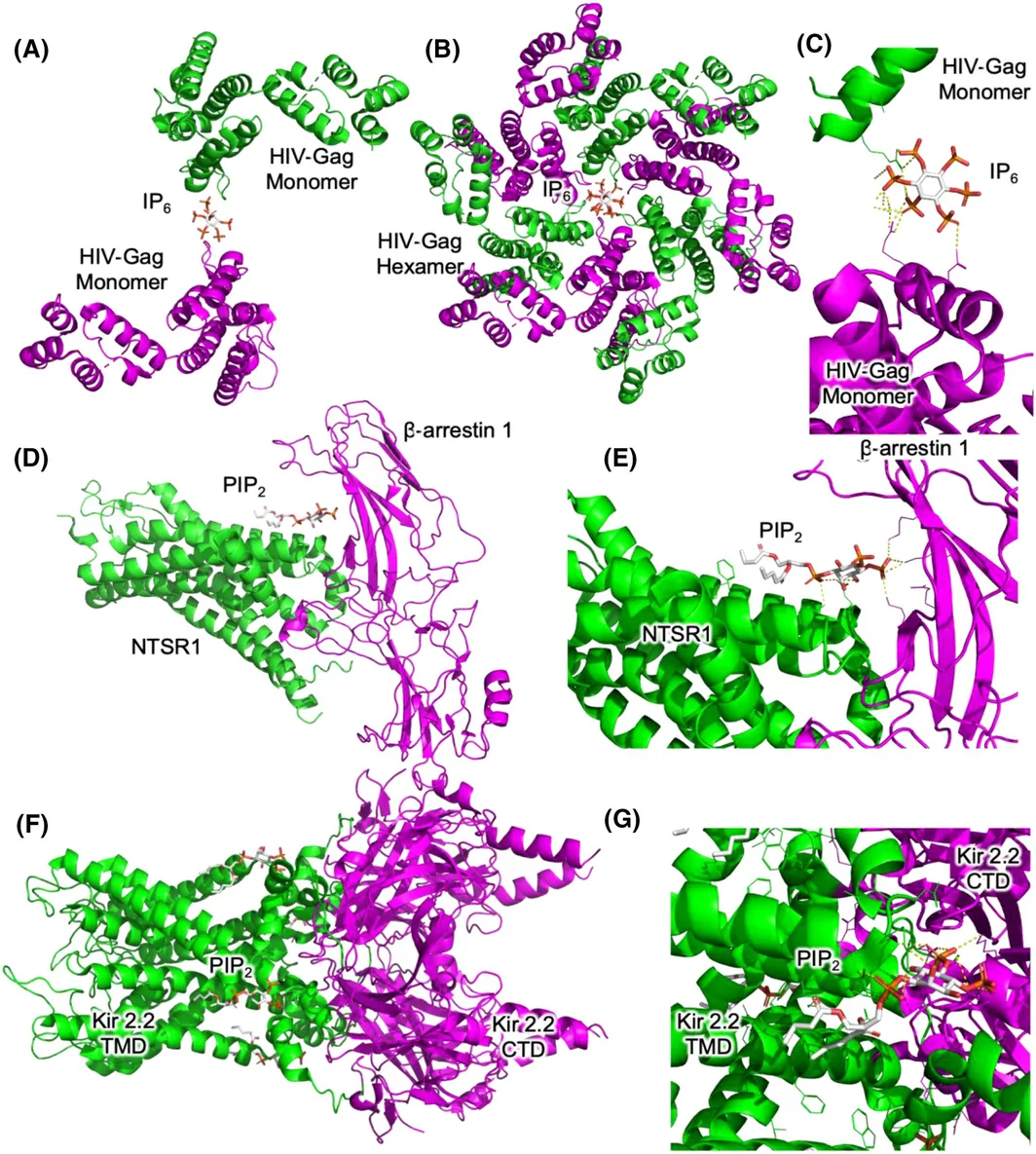
Both IP_6_ and PIP_2_ act as molecular glues for multiple complexes that are critical in signaling pathways. (A) Simplified representation of the HIV‐Gag hexamer interaction by two monomeric subunits (green and magenta) held together by IP_6_ (stick) (PDB: 6BHT), with (B) the complete complex (PDB: 6BHT), and (C) highlighting the ligand‐binding pocket with interactions shown with yellow dashed lines (PDB: 6BHT). (D) NTSR1 (green) interacting with β‐arrestin 1 (magenta) by PIP_2_ (stick) (PDB: 6UP7), with (E) highlighting the ligand‐binding pocket with interactions shown with yellow dashed lines (PDB: 6UP7). (F) The Kir 2.2 complex with the TMD (green) and CTD (magenta) glued by PIP_2_ (stick) (PDB: 3SPI), with (G) highlighting the ligand‐binding pocket with interactions shown with yellow dashed lines (PDB: 3SPI). These examples show the distinctive interactions by IP_6_ and PIP_2_ that are unique to each complex in their interaction interface in the proteins that are part of the complex, while maintaining their unique interaction mode.

As the virus matures into its final infectious form, the viral CA protein reorganizes into a fullerene‐like conical capsid, typically composed of 200–250 hexamers and 12 pentamers, which provide the curvature necessary for a closed shell [47, 48]. The stoichiometry of HIV‐Gag is such that each hexamer of Gag binds 1 molecule of IP_6_ [49], a stoichiometry shown to be critical for Gag structure and function [49]. There are 300–400 Gag hexamers per HIV virion [31], thus approximately 300–400 IP_6_ molecules per HIV virion that are incorporated into each virion as an essential structural component of the immature Gag lattice [31] (Fig. 5A–C). After budding, as the virus matures into its infectious form, IP_6_ continues to play a vital role by stabilizing the mature capsid. In this stage, IP_6_ binds near the R18 residues of the mature CA hexamers [31]. Although IP_6_ in this context could be viewed as an ‘assembly factor’ or ‘molecular architect’ of Gag assembly, it meets the definition of an endogenous molecular glue as it exists at the interface between proteins. Thus, IP_6_ serves as a molecular glue that is critical for proper immature lattice assembly and virion formation, two key stages of the HIV‐1 life cycle.

## IP_6_ in arrestin‐2 and arrestin‐3

Another example of an IPs acting as a molecular glue is found in the regulation of nonvisual arrestins. IP_6_ was shown to function as a molecular glue by facilitating the oligomerization of arrestin‐2 and arrestin‐3 [36]. The crystal structure of the arrestin‐2 IP_6_ complex revealed that IP_6_ binds to two distinct sites on arrestin‐2, enabling it to bridge adjacent arrestin molecules and promote oligomerization [36]. Biochemical assays and cellular studies confirmed IP_6_‐induced oligomerization, with mutations that disrupt binding sites impairing oligomerization without affecting other protein interactions [36]. Additionally, subcellular localization analyses showed that oligomeric arrestin‐2 resides in the cytoplasm, suggesting that IP_6_‐mediated oligomerization regulates the cellular distribution of arrestin and the function of arrestin by stabilizing the inactive, cytoplasmic forms [36].

## Phosphoinositides as molecular glues in signaling

IPs function as soluble signaling molecules, whereas PIPs, as membrane‐embedded lipids, most often recruit proteins to specific membrane sites. The distinct head groups of PIPs enable selective interactions that differ from those mediated by IPs, allowing PIPs to regulate pathways that are mechanistically distinct from those controlled by IPs [10, 50]. Well‐characterized examples include Kir2.2, an inward‐rectifying K^+^ channel activated by PIP_2_, and β‐arrestin, which is recruited to PIP_2_‐rich membranes to organize signaling complexes [34, 35].

A cryo‐EM structure (PDB: 6UP7) of the β‐arrestin 1 (βarr1) complexed with neurotensin receptor 1 (NTSR1) reveals how PIP_2_ acts as a crucial bridge to facilitate their interaction, which ultimately prevents G‐protein coupling and leads to GPCR internalization (Fig. 5D,E) [34]. This bridging interaction involves the C‐lobe groove's basic residues of βarr1 and the positively charged, membrane‐facing residues of NTSR1's TM1, TM2, and TM4 [34]. Specifically, the phosphates at positions 4 and 5 of PIP_2_ interact with R236, K250, K324, and K326 of βarr1 [34]. Evidence for PIP_2_'s essential role comes from experiments with a mutant βarr1 in the C‐ domain (K232Q/R236Q/K250Q) [34]. This mutation, which disrupts PIP_2_ complex formation, resulted in a 40% decrease in βarr1 binding to NTSR1 compared with the wild‐type [34]. These results establish PIP_2_ as a necessary role in the stable complex formation of β‐arr1 with NTSR1.

Another example of a phosphoinositide acting as an intramolecular glue is the inward‐rectifying Kir2.2 in complex with PIP_2_. X‐ray crystallography (PDB: 3SPI) reveals that PIP_2_ binds at the interface between the transmembrane domain (TMD) and the cytoplasmic domain (CTD) (Fig. 5F,G) [35]. The hydrophobic acyl chains of PIP_2_ interact with hydrophobic amino acids in the TMD helices, while the phosphates interact with an arginine‐tryptophan‐arginine sequence [35]. The phosphate of the PIP_2_ head group then forms contacts with K183, R186, K188, and K189, bridging the two domains [35]. This binding triggers a 6‐angstrom translation, tethering the CTD to the TMD and opening the inner ion channel gate, a mechanism analogous to neurotransmitter activation of neural synaptic ion channels.

**Fig. 6:**
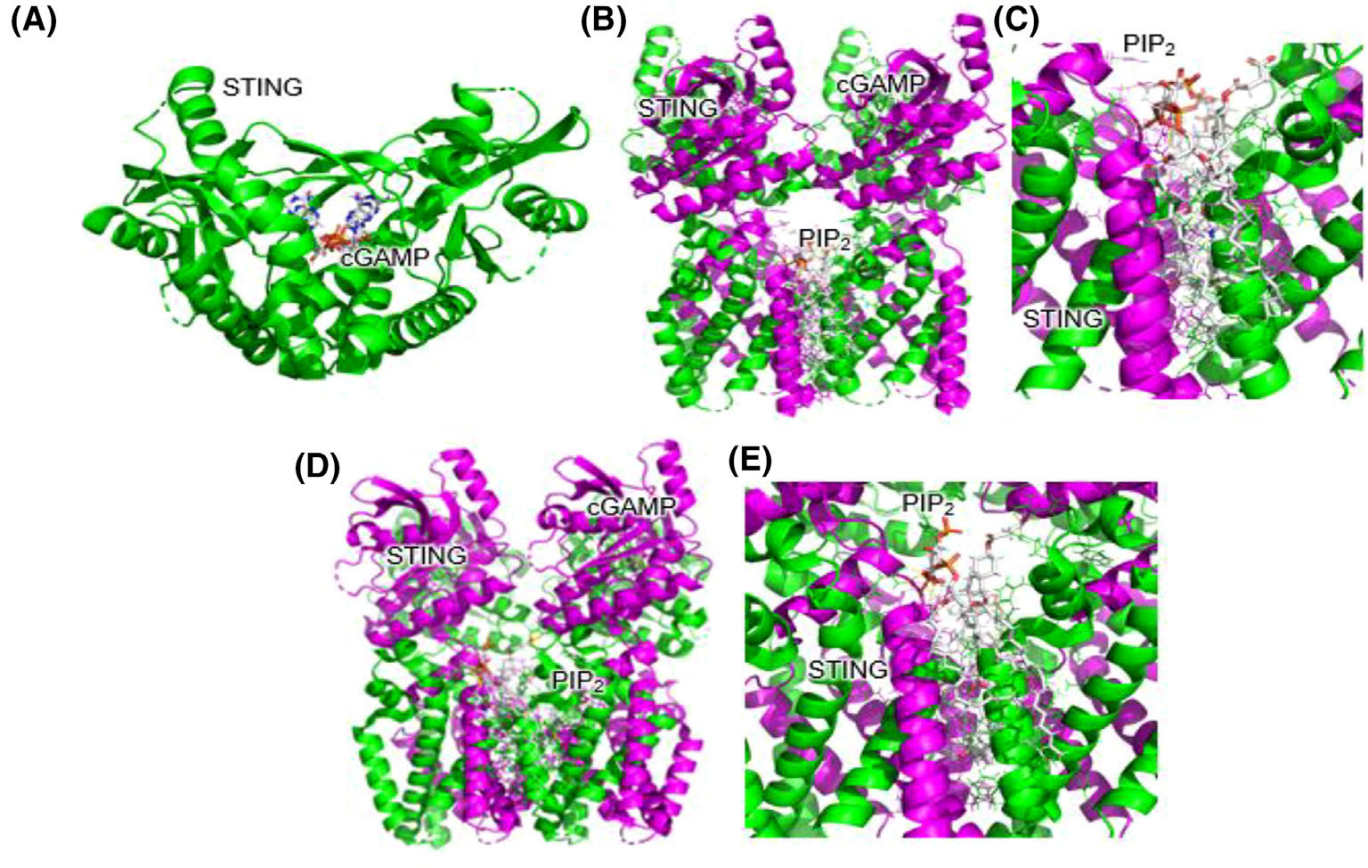
Nonligand‐binding pocket‐mediated glues: PIP_2_ acts as a molecular glue, mediating interactions of quaternary STING complexes. (A) The innate immune adapter protein STING (green) subunits glued by cGAMP (sticks) (PDB: 4KSY). (B) The tertiary complex of STING monomers (green and magenta), complexed with cGAMP (green sticks), and PIP_2_ (4,5) (white sticks) (PDB: 9DAN), (C) highlighting PIP_2_ binding to individual STING monomers. (D) The tertiary complex of STING monomers (green and magenta), complexed with cGAMP (green sticks), and PIP_2_ (3,5) (white sticks) (PDB: 9DAT), with (E) highlighting PIP_2_ binding to individual STING monomers. This shows that PIP_2_ acts as molecular glue without being bound to a traditional ligand‐binding pocket. Additionally, other molecules such as cGAMP are hypothesized to also serve as molecular glues, broadening the field of molecular glues.

More recently, PIP_2_ was shown to mediate oligomerization of the essential DNA‐sensing adaptor in innate immunity stimulator of interferon genes (STING), in response to cGAMP (PDB: 4KSY) (Fig. 6A) [37]. Cryo‐EM structures presented in the same paper show PI(3,5)P_2_ (PDB: 9DAT) and PI(4,5)P_2_ (PDB: 9DAN) exist directly in the interface between the STING oligomers, strongly suggesting a molecular glue function for these two distinct PIP_2_ molecules (Fig. 6B–E) [37].

## Pseudo‐glue‐like interactions and those awaiting further investigation

The distinctive instances of molecular glue previously described are clear in their mode of interaction and the functional consequences of that interaction. It should be noted that some interactions have been categorized as molecular glues, yet do not fit our strict definition. The first example is PIP_3_ in the nuclear receptor SF‐1, which creates a novel protein–lipid interaction surface as shown by multiple biophysical techniques and structural investigations [14]; however, identification of those binding partners is still under investigation. Binding to SF‐1 with a high affinity (80 nM), PIP_3_ binding also significantly increases the thermal stability of SF‐1. X‐ray crystallography (PDB: 4QJR for PIP_3_‐bound SF‐1 and PDB: 4QK4 for PIP_2_‐bound SF‐1) revealed the binding topology, with PIP_3_'s hydrophobic tails embedded within SF‐1's ligand‐binding pocket, while its charged head group is fully solvent‐exposed at the pocket's entrance [14]. This arrangement organizes previously disordered dynamic surface loops (L2‐3 and L11‐12) into a structured activation function surface, which is crucial for coactivator binding. Further analyses were conducted on this example and showed striking differences comparing the SF‐1/PIP_3_ structure with the LRH‐1/DAX‐1 corepressor complex. These data suggested that the exposed PIP_3_ headgroup sterically clashes with the DAX‐1 binding site, indicating that PIP_3_ binding not only activates SF‐1 but also prevents corepressor binding, thereby locking SF‐1 in an active conformation and facilitating productive coactivator interactions essential for transcription regulation. In addition to DAX‐1, it is also proposed that pleckstrin‐homology (PH)‐domains, such as that of phosphoinositide‐dependent kinase‐1 (PDK1), can bind to the exposed head group of PIP_3_ when bound to SF‐1 [51, 52]. This is further supported by inositol lipid kinase IPMK being shown to bind PIP_2_ while bound to SF‐1 [53, 54].

A second example of a pseudo‐glue is inositol pyrophosphates IP_7_ (isomer 5‐PP‐IP_5_) and IP_8_ in the phosphate‐sensing and acquisition (PHO) pathway. This pathway involves the kinase PHO85, the cyclin PHO80, and the CDK inhibitor PHO81, which regulate transcriptional responses that maintain phosphate homeostasis [38]. Affinity capture experiments confirmed that PHO81 is associated with IP_7_ via specific lysine residues (K221, K224, and K228) in its SYG1/PHO81/XPR1 (SPX) domain [38]. While the PHO81‐PHO85‐PHO80 complex forms regardless of phosphate levels, PHO81 was identified as the most phosphate‐responsive component [55]. Importantly, in an IP_7_‐deficient environment, PHO81 degrades while PHO85 does not, suggesting that IP_7_ binding is essential for PHO81 stability and its association with the PHO85‐PHO80 complex, promoting PHO pathway activity [38]. Previous studies from Oshea et al. indicate a more complex role for IP_7_. While IP_7_ is required for PHO81‐dependent inhibition of PHO80‐PHO85, research has shown that the ‘minimal domain’ (MD) of PHO81, rather than the SPX domain, governs the association of PHO81/IP_7_ with PHO85‐PHO80. IP_7_ interacts noncovalently with PHO80‐PHO85‐PHO81 and induces additional interactions between PHO81 and PHO80‐PHO85, thereby preventing substrates from accessing the kinase active site [55].

While the classical model of the PHO pathway suggested that an increase in IP_7_ serves as the primary trigger for PHO85‐PHO80 inhibition through the PHO81 minimal domain, recent evidence using full‐length proteins and isomer‐specific analysis provides a revised regulatory framework [38, 39]. Under this updated model, inositol pyrophosphates such as IP_7_ and IP_8_ significantly decline upon phosphate starvation [38]. Specifically, IP_7_ is characterized as an intermolecular ‘glue’ that stabilizes the association between the PHO81 SPX domain and the PHO80 cyclin [38]. This structural stabilization is essential for protein integrity; without IP_7_ binding to the conserved lysine surface cluster of the SPX domain, the PHO81 inhibitor becomes unstable and is rapidly degraded *in vivo* [38]. Consequently, the activation of the PHO pathway is not triggered by an IP_7_ increase, but rather by the depletion of IP_8_, which serves as a physiological switch that allows the stabilized PHO81‐PHO85‐PHO80 complex to transition into an active inhibitory state [56]. This framework helps explain the vip1Δ yeast strains which cannot synthesize IP_8_. In these mutants, the PHO pathway fails to activate because the absence of IP_8_ prevents the inhibitory complex from ever properly assembling. Essentially, if the IP_8_ is missing, PHO81 cannot stabilize its interaction with the PHO80‐PHO85 kinase; the kinase therefore remains active and keeps PHO4 sequestered in the cytoplasm [38, 39].

Moreover, the role of IP_7_ extends beyond stabilization, as it can also disrupt interactions within the SPX domain, exemplified by its effect on the VTC complex [57]. In the VTC complex, IP_7_ binding to the SPX domains of VTC2 and VTC3 disrupts homotypic SPX–SPX interactions, activating the complex. This suggests IP_7_ functions destabilize this protein complex [58]. Taken together, these are examples of IP_7_ as a pseudo‐glue, as while IP_7_ can act to stabilize the PHO81‐PHO85‐PHO80 complex, more work is required to establish whether it exists in these protein–protein interfaces. In these systems, IP_7_ can also induce conformational rearrangements or disrupt existing interactions in other SPX‐containing complexes. Thus, IP_7_ exhibits a multifaceted, context‐dependent mode of action that encompasses both stabilizing and destabilizing effects on protein assemblies.

Similarly, the multidomain adaptor protein 2 complex (AP2) and the heterotetrametric clathrin adaptor complex are known to bind to IPs and PIPs. Structural analysis of the IP_6_‐bound AP2 complex reveals that the IPs do not reside directly within the protein–protein interface. While IP_6_ is essential for complex formation and enhances the structural stability of the assembly, its binding at a site distal to the interaction surface classifies it as a pseudo‐glue—rather than a true molecular glue [59]. The stoichiometry of IP_6_ to AP2 is 1:1, with each IP_6_ molecule making contact with two separate parts of two different AP2s [59]. The binding of IP_6_ induces a conformational change that allows AP2s to bind to ligands, aiding the process of assembly and disassembly of clathrin‐mediated vesicles [59].

In all these instances, IPs or PIPs are either in the correct structural position to act as a molecular glue, or functionally induce a protein–protein interaction; however, further research will be required to establish that both these requirements of a molecular glue are met in these systems.

## Formation and reversal of molecular glues

In order to have regulatory value to the cell, molecular glue interactions must be dynamic and able to be governed by regulatory mechanisms that enable rapid assembly and disassembly in response to cellular signals. The assembly and disassembly of these complexes formed by IPs and PIPs are dictated by three main factors: (i) ligand concentration, which drives the equilibrium toward ternary complex formation [60, 61]; (ii) enzymatic modification where kinases, phosphatases, and lipases alter the affinity of the molecular glue for the interface [30]; (iii) and subcellular location, which dictates local availability of IPs and PIPs through diffusion or membrane sequestration [11, 12, 13, 17].

The assembly of molecular glue is a concentration‐dependent equilibrium process; each ligand must reach thresholds that exceed the dissociation constant (K_d_) required at the protein–protein interface [60]. This is represented by the assembly of the immature HIV‐1 Gag lattice and the mature viral capsid, which depends on the recruitment of IP_6_ to the hexameric interface. Without a sufficient local concentration of IP_6_, the structural integrity of the virus is not maintained [31, 45, 61].

Conversely, disassembly of molecular glue‐based complexes is often achieved through enzymatic turnover, where signaling enzymes alter the effective concentrations of the molecular glue. A classic example is phospholipase C (PLC) hydrolysis of PIP_2_ into diacylglycerol and IP_3_. The resulting decrease in PIP_2_ disrupts PIP_2_‐dependent recruitment and stabilization of protein–protein interactions, such as the CTD and TMD of Kir2.2 channels (Fig. 5G) [35, 62].

Together, these mechanisms emphasize that molecular glues can function as reversible, regulated interfacial molecules, rather than permanent adhesive elements, providing context‐dependent modulation of protein–protein interactions.

## Conclusions

In this review, we detailed the emerging roles of IPs and PIPs as molecular glues that coordinate complex signaling and cellular functions. While these signaling molecules have established modes of action as traditional allosteric modulators of protein function, their less well‐appreciated functions as molecular glues reviewed here represent a distinct but growing mode of action that is being more appreciated for important effects on cellular homeostasis [53]. The take‐home message for future research is that the molecular glue mode of action is distinct from allostery: in these cases, the small molecule binds at an allosteric site distinct from the protein–protein interface, triggering a shape change that only indirectly facilitates recruitment. By defining IPs and PIPs as true molecular glues, we recognize them not just as transient messengers in signal transduction, but as the physical interactions that hold together two proteins (or protein domains) to stabilize the interaction. The molecular glue mode of action described here is not unique to IPs or PIPs; examples illustrated herein are the plant hormone auxin [63] and adenosine monophosphate [28] which can both be defined as molecular glues, but of course are not inositol‐based molecules. Perhaps the most significant gap that is required to identify more molecular glues is the structural description required.

Being able to distinguish molecular glue vs. allosteric mechanisms of action is essential for properly targeting drug screening and other development efforts to maximize clinical and therapeutic impact. Effective drug screening and development for an allosteric modulator must be inherently distinct from that of a molecular glue, if it is to be effective. Thus, the impact of understanding molecular glue‐mediated events has potential as a vast, untapped frontier in drug discovery. Ultimately, characterizing the molecular glue functions of IPs and PIPs will provide us with a more accurate understanding of how these molecules mediate physiology and signaling in the cell, and will aid in the discovery and development of novel therapeutics. Without this understanding, we lose the complete picture of how these highly abundant, well‐conserved molecules mediate their biological functions.

## Author contributions

AST conceptualized the project, drafted and edited the manuscript, and wrote the manuscript. AJC conceptualized the project, drafted and edited the manuscript, and prepared structural figures. RDB conceptualized the project and edited the manuscript. MNCL, JM, JG, JK, MA, STP, and DZ conceptualized the project.
